# Psychometric properties of Structured Clinical Interview for DSM‐5 Disorders‐Clinician Version (SCID‐5‐CV)

**DOI:** 10.1002/brb3.1894

**Published:** 2021-03-17

**Authors:** Amir Shabani, Samira Masoumian, Somayeh Zamirinejad, Maryam Hejri, Tahereh Pirmorad, Hooman Yaghmaeezadeh

**Affiliations:** ^1^ Mental Health Research Center Mood Disorders Research Group Iran University of Medical Sciences Tehran Iran; ^2^ Clinical Psychology Department School of Behavioral Sciences and Mental Health (Tehran Psychiatry Institute) Mental Health Research Center Iran University of Medical Sciences Tehran Iran

**Keywords:** psychiatric disorders, reliability, validity

## Abstract

**Objectives:**

The purpose of this study was to evaluate the psychometric properties of Structured Clinical Interview Version for DSM‐5 (R) Clinical Version (SCID‐5‐CV) in a population of patients with psychiatric disorders in Tehran.

**Method:**

The study population included all outpatients and inpatients referred to three psychiatric centers in Tehran, namely Iran Psychiatric Hospital, Rasoul Akram Hospital, and Clinic of Behavioral Sciences and Mental Health (Tehran Psychiatric Institute). Inclusion criteria included age between 16 and 70 years, informed consent to study, ability to understand and speak Persian, and no specific physical problems that interfere with the conduct of the interview. Also, exclusion criteria included inability to communicate, mental retardation or dementia, severe symptoms of acute psychosis, and severe restlessness. In addition to demographic questionnaire, Persian version of SCID‐5‐CV was used in this study. Finally, diagnostic validity, test–retest reliability, and inter‐rater reliability were used to evaluate the information.

**Results:**

In terms of the kappa criterion, for all diagnoses except for anxiety disorders, kappa was above 0.4 as a result of agreement above average, but in anxiety disorders with kappa 0.34 there was a moderate agreement between psychiatrist and SCID interviewer reports. Also, according to the psychiatrist's diagnosis as the gold standard, in most diagnoses, except for anxiety disorders, kappa was higher than 0.80, indicating the desirable characteristic of this tool in the diagnosis of disorders. Sensitivity of all diagnoses was higher than 0.80.

**Conclusion:**

According to the findings of the present study, SCID‐5‐CV can be used for diagnostic purposes in psychiatric clinics and hospitals and to evaluate the treatment process of patients. In general, this version is suitable especially the schizophrenia spectrum and other psychiatric disorders; however, using SCID‐5‐CV for anxiety‐related disorders should be done with caution.

## Graphical abstract

SCID‐5‐CV can be used for diagnostic purposes in psychiatric clinics and hospitals and to evaluate the treatment process of patients. This version is suitable especially the schizophrenia spectrum and other psychiatric disorders

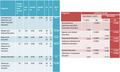

## 1 INTRODUCTION

Since the publication of the first edition of the Diagnostic and Statistical Manual of Mental Disorders (DSM), most of the diagnoses have been under question. Since diagnoses are usually not based on a unified standard model, the possibility of all other diagnoses is not exhausted; thus, the assessment is not comprehensive; consequently, any diagnosis is highly dependent on the experience and performance of the diagnostician, leading to a lower reliability (Steiner et al., 1995). Therefore, structured interviews are devised to collect information and evaluate the symptoms in a definite and comprehensive manner, and interviews are performed using a standard algorithm to make diagnoses more accurate and reliable (Sommers‐Flanagan & Sommers‐Flanagan).

Structured Clinical Interview for the DSM (SCID) is a semistructured interview that provides diagnoses based on DSM. It requires the interviewer's clinical judgment about the interviewee's responses, and therefore, the interviewer must have the knowledge and clinical experience in the field of psychopathology and DSM classifications and diagnostic criteria. One of the goals of its creators was the simplicity of the procedure, while enjoying a structured framework (Spitzer et al., 1992). The Structured Clinical Interview for DSM‐5 (SCID‐5) (First et^2017^l.,^2017^), which is based on the latest version of DSM, has five manuals: SCID‐5‐CV (Clinician Version) (First et al.,2015a), SCID‐5‐RV (Research Version) (First et al.,2015b), SCID‐5‐CT (Clinical Trials Version) (First et al.,^2014^), SCID‐5‐PD (Personality Disorders) (First et al., 1997), and Alternative Model for Personality Disorders (SCID‐5‐AMPD) (First et al.).

Structured diagnostic interviews come handy in many spheres of psychology and psychiatry. First, clinical researchers should be able to determine whether study participants meet the inclusion or exclusion criteria. Second, in clinical practice, specialists often encounter different scenarios and they should be able to evaluate patients on a clear diagnostic criterion. Third, training programs often use structured diagnostic interviews to teach the interview process and familiarize trainees with diagnostic criteria (Tolin et al.).

The DSM‐5 made several significant changes to the Diagnostic and Statistical Manual of Mental Disorders (DSM‐IV). These changes can generally be observed at three levels: (a) general level: dimensional pattern rather than categorical pattern and elimination of the multi‐axis system; (b) interclass level: (1) taking out parts of disorders from a previous diagnostic class, such as obsessive–compulsive disorder or trauma and stress disorder and (2) new disorders such as Hoarding disorder; (c) intraclass level: changes in criteria for specific disorders and reduction or addition of criteria or part of existing criteria (Association AP). Therefore, a structured diagnostic interview based on the DSM‐5 diagnostic criteria seemed necessary.

Before SCID‐5, it is noteworthy that various studies have shown the favorable validity and reliability of Structured Clinical Interview for DSM‐ IV (SCID‐IV) and Structured Clinical Interview for DSM‐III‐R(SCID) (Lobbestael et al., 2011; Martin et al., 2000; Segal et al., 1993, 1995; Sharifi et al., 2004; sharifi Vea, 2017; Skre et al., 1991; Torrens et al., 2004; Williams et al., 1992; Zanarini & Frankenburg, 2001; Zanarini et al., 2000). However, to date, limited psychometric data have been published for SCID‐5.

In examining the psychometric properties of structured interviews based on SCID‐5, most work has focused on interviews with specific disorders, such as anxiety disorders, PTSD, or alcohol and substance use disorders. The most comprehensive of them is the work done by Tolin et al who studied the psychometric properties of the SCID‐5 structured interview for anxiety, mood, Obsessive‐Compulsive and Related Disorders (DIAMOND). 362 adult patients underwent DIAMOND interview. The data of 121 patients gave inter‐rater reliability and 115 of them provided the test–retest data. Reliability between DIAMOND raters ranged between very good and excellent (*κ* = 0.62 to *κ* = 1.00) and DIAMOND test–retest reliability between good and excellent (*κ* = 59 to *κ* = 1.00) (Tolin et al.,). There has been no study on the validity and reliability of SCID‐5 structured interviews in Iran. But, the validity and reliability of SCID‐IV in a study by Sharifi et al have been investigated in two stages: (a) manual translation and its cross‐cultural validity analysis, including direct and reverse translation and content validity according to intercultural indicators; (b) study of reliability and applicability of the instrument in Iranian clinical population. In this study, 299 participants aged 18–65 years referred to outpatient clinics and inpatient wards of three psychiatric centers of Roozbeh Psychiatric Hospital (Tehran University of Medical Sciences), Imam Hossein Hospital Psychiatric Complex (Shahid Beheshti University of Medical Sciences), and Iran Psychiatric Hospital (Iran University of Medical Sciences). In the test–retest reliability study, 104 clients were independently evaluated with SCID‐5 on two visits (three to seven days apart). Feasibility was assessed by interviewees (299) and interviewers by questionnaires that included questions about the duration of the interview, how boring it was, its lucidity and acceptability of the questions, and how much effort it needed. Findings showed that diagnostic agreement was moderate to good for most specific and overall diagnoses (*κ* > 0.6). The overall agreement (total kappa) was 0.52 for all current diagnoses and 0.55 for lifetime diagnoses. Most interviewees and interviewers find the SCID version of Farsi acceptable (Sharifi et al., 2004).

Obviously, the translation of an interview is not enough for its use in another culture and special attention should be paid to interlinguistic and intercultural differences in order to maintain its validity. In addition, the reliability and validity of the translated tool in the target culture should be measured and thus standardized. As far as the present researchers are aware, the psychometric properties of none of the diagnostic interviews based on SCID‐5 have so far been studied in Iran, and since these structured interviews form the basis of most of the therapeutic and research work in psychology and psychiatry, the purpose of this study was to investigate the psychometric properties of the Persian version of SCID‐5‐CV.

## 2 METHOD

The study population is comprised of all outpatients and inpatients admitted to three psychiatric centers in Tehran, namely Iran Psychiatric Hospital, Rasoul Akram Hospital, and Clinic of Behavioral Sciences and Mental Health (Tehran Psychiatric Institute). A total of 250 patients were recruited. In order to evaluate the test–retest reliability, 106 patients were interviewed after an interval of 7–10 days. Inclusion criteria were being 16–70 years of age, being able to understand and speak Persian, and having no specific physical problems that can interfere in the interview. Also, exclusion criteria were severe irritability, mental retardation, and dementia, as well as acute psychosis to the extent that they were unable to participate in the interview. Following the proper authorization from the ethics committee of the Iran University of Medical Sciences and coordination with the mentioned centers, patients who met the inclusion criteria were invited for interview. Informed consent was acquired, and their rights were explained to them including the freedom to discontinue at any stage of the research. The interviews were conducted privately and without access to the patients' records. Outpatient's interviews were conducted as they were waiting in the hospital premises, and inpatients interviews were conducted during the first week of their stay. Interviewers were carried out by Ph.D. students in clinical psychology. Interviewers were provided with a summary of the hospital admission sheet for inpatients and a report of their first visit for outpatients. This information was only available to those researchers who were not involved in the interview process, and the interviewers were not aware of their interviewees’ diagnoses. One of the two interviewers in each room did the interview before both of them made their diagnoses. The gold standard of diagnosis was the records in the hospital/clinic files according to the routine standards of these university‐affiliated hospitals/clinic. This routine include (a) early interview with the patient by a resident of Psychiatry, (b) gathering the history data of patient including lifetime course of the disorder, and any previous treatment and recorded psychiatric diagnosis in outpatient and inpatient settings, (c) interview with accessible family members, and (d) recording the final diagnosis by a supervisor Psychiatrist based on an independent interview with the patient and all the gathered data, according to the DSM‐5.

## 3 RESEARCH TOOLS

- Demographic Questionnaire: Personal information questionnaire about sex, age, level of education, marital status, number of children, history of psychological disorders, and history of suicide attempts.
- Structured clinical interview for DSM‐5 disorders‐clinician version (SCID‐5‐CV): The SCID‐5‐CV is a comprehensive standardized tool for evaluating major psychiatric disorders based on DSM‐5 definitions and criteria. According to DSM‐5, diagnoses categories include schizophrenia spectrum and other psychiatric disorders, bipolar and related disorders, depressive disorders, substance‐related and addictive disorders, anxiety disorders, obsessive–compulsive and related disorders, post‐traumatic stress disorder, attention‐deficit/hyperactivity disorder, and other disorders. This interview is designed for clinical and research purposes. SCID‐5‐CV is usually implemented in one run which takes between 45 and 90 min (Sharifi et al., 2017)

## 4 RESULT

This study is a descriptive correlational study. The population was consisted of all outpatient and inpatients referred to the three psychiatric centers in Tehran. Overall, data from 245 patients were analyzed, of whom 105 (42.9%) were male and 140 (57.1%) were female. The age ranged from 17 to 68 years with a mean of 35.91 and standard deviation of 11.64. Table 1 shows the demographic characteristics of participants.

**Table 1:** Frequency and percentage for demographic characteristics

Variable	Subcategory	Frequency	Percentage
Gender	Male	105	42.9
	female	140	57.1
marital status	Single	107	43.7
	Married	101	41.2
	divorced	34	13.9
	Widow	3	1.2
Education status	Elementary or middle school	98	40
	Diploma	72	29.4
	Associate	23	9.4
	Bachelor	30	12.2
	Masters and higher	20	8.2
Employment status	Unemployed	78	31.8
	Self‐employed	81	33.1
	Employee	20	8.2
	House wives	39	15.9
	Student	18	7.3
	Retired	9	3.7
Suicidal thoughts and behavior(Lifetime)	Lack of thought and behavior	105	42.9
	Suicide attempt	77	31.4
	Suicidal thoughts	63	25.7
Drug or alcohol abuse(Lifetime)		54	22
Serious physical illness		91	37.1
History of treatment	Hospitalization	137	55.9
	pharmacological	171	69.8
	Psychotherapy	10	4
	pharmacological and Psychotherapy	12	4.9

The structured clinical interview for DSM‐5 (R) clinical version (SCID‐5‐CV) translated by Sharifi et al. (2017) was used for validity and reliability study.

The whole sample (*n* = 245) was initially evaluated by psychiatrists according to DSM‐5 criteria. Five interviewers, clinical psychology Ph.D. students implemented the SCID‐5‐CV without the knowledge of a psychiatrist diagnosis.

**Figure 1:**
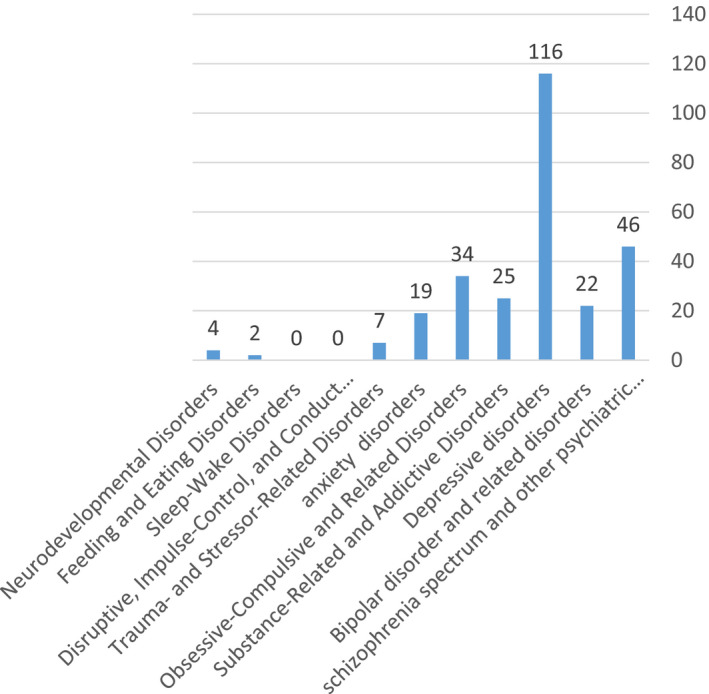
Frequency of psychiatry disorders

Figure 1 shows frequency of psychiatry disorders in this study.

Test–retest reliability was assessed by one of the interviewers through visiting 113 (46.1%) patients after 7–10 days. As seen in Chart 1, the prevalence of depressive disorders was the highest. Some of group of disorders (trauma and stressor‐related disorders, sleep–wake disorders, eating disorders, maladaptive disorders, impulse control disorders, conduct disorder, and neurodevelopmental disorders) were excluded due to low frequency.

Table 2 shows the rate of agreement between SCID and psychiatrists (Kappa criteria), as well as the sensitivity, specificity, positive, and negative likelihood ratios, and LR+/LR ratio of these instruments if the psychiatrist's diagnosis is considered as the gold standard. As can be seen in this table, kappa was above 0.4 for all diagnoses except for anxiety disorders. The schizophrenia spectrum and other psychiatric disorders with a kappa of 0.90 reflect the almost complete agreement between the psychiatric reports and the SCID interviewer. In Bipolar and related disorders, depressive disorders, substance‐related and addictive disorders, and obsessive–compulsive disorder, the kappa ranged from 0.76 to 0.80 reflecting the high agreement between the psychiatric reports and the SCID interviewer. Only anxiety disorders with a kappa of 0.34 indicate moderate agreement between the psychiatric reports and the SCID interviewer.

**Table 2:** SCID and psychiatrists' diagnoses agreement (based on DSM‐5 diagnostic criteria) and sensitivity, specificity, and positive and negative likelihood ratio (*n* = 245)

LR+/LR−	Diagnosis	Frequency (By Gold standard)	Kappa	sensitivity	specificity	LR+	LR−
404.5	Schizophrenia spectrum and other psychotic disorders	46	0.9	0.89	0.98	44.5	0.11
113.88	Bipolar and related disorders	22	0.8	0.82	0.96	20.5	0.18
27.63	Depressive disorders	116	0.69	0.84	0.84	5.25	0.19
183.33	Substance‐related and addictive disorders	25	0.76	0.88	0.96	22	0.12
61	Anxiety disorders	19	0.34	0.94	0.78	4.27	0.07
113.88	Obsessive–compulsive disorder	34	0.76	0.82	0.96	20.5	0.18

If the diagnoses provided by psychiatrists were considered to be the gold standard, the specificity results were generally better than the sensitivity results, meaning the in most of the diagnoses except for anxiety disorders, they were above 0.80, indicating the desirable specificity. The sensitivity of all diagnoses was higher than 0.80. LR+/LR− ratios also showed that this tool made the best diagnosis for the schizophrenia spectrum and other Psychotic Disorders. It also has the potential to be useful for bipolar and related disorders, substance‐related and addictive disorders, anxiety disorders, and obsessive–compulsive disorder, but it would not be desirable for depressive disorders.

To assess the inter‐rater reliability, two examiners completed the interviews separately. The phi coefficients (Table 3) showed that in all diagnoses, there is a very strong correlation at *α* < 0.0001 significance level. Therefore, the SCID‐5‐CV has very good inter‐rater reliability. In addition, 113 patients were interviewed to assess the test–retest reliability. The results of the first and second interview coefficients of phi showed that there is a strong relationship between first and second interviews with *α* < 0.0001 in case of obsessive–compulsive disorder. There was also a significant relationship between the schizophrenia spectrum and other Psychotic Disorders, bipolar and related disorders, substance‐related and addictive disorders, and anxiety disorders at *α* < 0.0001. However, a coefficient of 0.397 with a significance level of *α* < 0.0001 in depressive disorders showed that although there is a significant relationship between the first and second interviewers, this relationship is very weak. Therefore, SCID‐5‐CV has good test–retest reliability in all diagnostic disorders except for depressive disorders.

**Table 3:** Phi coefficients of SCID diagnoses

Diagnosis	coefficient of phi			
	Interviewer 1 and 2		Test‐retest	
	*C* value	sig	*C* value	sig
Schizophrenia spectrum and other psychiatric disorders	0.986	>0.001	0.508	>0.001
Bipolar and related disorders	1	>0.001	0.598	>0.001
Depressive disorders	0.951	>0.001	0.397	>0.001
Substance‐related and addictive disorders	0.896	>0.001	0.412	>0.001
Anxiety disorders	0.916	>0.001	0.488	>0.001
Obsessive–compulsive disorder	0.951	>0.001	0.774	>0.001

## 5 DISCUSSION

The purpose of this study was to evaluate the psychometric properties of Structured Clinical Interview for DSM‐5 (R) Clinical Version (SCID‐5‐CV) in 245 patients with psychiatric disorders in Tehran. Kappa was above 0.4 for all diagnoses except for anxiety disorders. The schizophrenia spectrum and other psychiatric disorders with a kappa of 0.90 indicate almost complete agreement between the gold standard diagnosis and that of the SCID interviewer. Bipolar and related disorders, depressive disorders substance‐related and addictive disorders, and obsessive–compulsive disorder and related disorders with a kappa of ranging from 0.76 to 0.80 indicate high agreement between the two diagnoses. Only anxiety disorders with a kappa of 0.34 indicate moderate agreement. Overall, the results indicate a high diagnostic agreement between the SCID interview and the gold standard diagnosis. Only for anxiety disorders does the agreement seem to be moderate.

Our findings are somewhat in accordance with the results of another researches (Amini et al., 2007; Sharifi et al., 2004) that examined the validity of Structured Diagnostic Interview for Axis 1 Disorders. They found the lowest agreement for lifetime diagnoses of SCID and psychiatrists with anxiety disorders and the highest agreement for the schizophrenia spectrum and other psychiatric disorders. However, the level of agreement between the diagnoses of SCID and the gold standard diagnosis in the present study was higher than previous studies. Shankman et al. found the lowest agreement for social anxiety and agoraphobia (Shankman et al.).

If the psychiatrist's diagnosis is considered to be the gold standard, the specificity and sensitivity of the diagnoses are high, and in most cases, except for anxiety disorders, the specificity was higher than the sensitivity. This indicates that the false‐positive rate of the given diagnoses is low. These findings are in line with the results of Amini et al. (2007) and Sharifi et al. (2009). But unlike those studies that found sensitivity indices in most diagnoses to be somewhat low (between 60% and 80%) and concluded that this tool cannot be used for large epidemiological studies, the present study provides good sensitivity for SCID 5‐CV and provides applicability for large epidemiological studies.

Examination of the LR+/LR− ratio showed that this interview made the best diagnosis for the schizophrenia spectrum and other psychiatric disorders. It also has the potential to be useful for bipolar and related disorders, substance‐related and addictive disorders, anxiety, and obsessive–compulsive disorders and related disorders, but will be weaker for depressive disorders than other diagnoses. These results have the highest utility for the schizophrenia spectrum and other psychiatric disorders and the lowest for mood and anxiety disorders, which is consistent with the results of Amini et al. (2007) and Sharifi et al. (2009), but overall the LR+/LR− were higher than previous studies.

Moreover, there was a strong correlation between the first and second interviewers in all diagnoses at a significance level of *α* < 0.0001, indicating a very good Reliability. Also, a review of the LR+/LR− ratio showed that this tool made the best diagnosis for the schizophrenia spectrum and other psychiatric disorders. It also has the potential to be useful for bipolar and related disorders, substance‐related and addictive disorders, anxiety disorders, and obsessive–compulsive disorder, but it will be weaker for depressive disorders. These results have the highest utility for the spectrum of schizophrenia and other psychotic disorders and the lowest for mood and anxiety disorders, that is consistent with the results of Amini et al. (2007) and Sharifi et al. (2009), but overall the LR+/LR− ratio were higher than previous studies. In addition, SCID‐5‐CV shows a good inter‐rater reliability for all diagnoses. In this respect, the present study is in line with the results of Lobbestael et al. (2011). The test–retest reliability results for all diagnoses except for depressive disorders confirm the validity of SCID‐5‐CV.

As with any research, the present study has some limitations that will hurdle the generalization and reliance of the findings. The participants of the present study were limited to a specific geographic area, with a limited number of voluntary and purpose‐based. Also, the limited sample size and different frequency of diagnoses occurrences and prevalence were other shortcomings of the study. Any generalization thus should be done with due caution.

Therefore, it is suggested that similar research be done on more various samples with different demographic characteristics in different cultures. Also, it can be suggested that clinical practitioners can use this version well in their research and treatment, but caution should be exercised regarding depression and anxiety disorders. Lower frequencies of some disorders and consequently insufficient diagnostic classes, that forced out five classes of disorders (trauma and stressor‐related disorders, sleep disorders, eating disorders, disruptive disorders, impulse control disorders, and neurodevelopmental disorders), necessitate that further research from different centers be done to study these disorders and to validate the Persian version of SCID‐5‐CV.

## 6 CONCLUSION

Overall, the acceptable reliability and validity of the SCID‐5‐CV diagnoses showed that the Persian version of the SCID‐5‐CV was a valid and reliable instrument for diagnoses. It can be used for clinical, research, and educational purposes and is suitable for most diagnoses, especially the schizophrenia spectrum and other psychiatric disorders. Only with regard to the diagnoses received for anxiety disorders should this be used more carefully. Therefore, the researchers recommend using this interview as a diagnostic aid in clinical settings.

## CONFLICT OF INTEREST

The authors declare that they no competing interests.

## AUTHOR CONTRIBUTIONS

Dr Amir Shabani conceptualized and designed the study and drafted the manuscript. Dr Samira Masoumian designed the study, collected the data, and drafted the manuscript. Dr Somayeh Zamirinejad, Maryam Hejri, and Tahereh Pirmorad collected the data. Hooman Yaghmaie Zadeh analyzed and interpreted the data and involved in statistical analysis.

### PEER REVIEW

The peer review history for this article is available at https://publons.com/publon/10.1002/brb3.1894.

## Data Availability

The data that support the findings of this study are available from the corresponding author upon reasonable request.
